# The Effects of a Ball Combination Training Program Combined with a Continuous Theta Burst Stimulation Intervention on Eating Behaviors in Autistic Children with Accompanying Intellectual Disabilities: A Preliminary Study

**DOI:** 10.3390/nu17091446

**Published:** 2025-04-25

**Authors:** Yufei Liu, Kelong Cai, Kai Qi, Xuan Xiong, Zhiyuan Sun, Yifan Shi, Aiguo Chen

**Affiliations:** 1 Gdansk University of Physical Education and Sport, 80-336 Gdansk, Poland; yufei.liu@awf.gda.pl (Y.L.); kai.qi@awf.gda.pl (K.Q.); 2 College of Physical Education, Yangzhou University, Yangzhou 225127, China; mx120170353@yzu.edu.cn (K.C.); dx120220089@stu.yzu.edu.cn (Z.S.); dx120220088@stu.yzu.edu.cn (Y.S.); 3 Department of Physical Education, Nanjing University, Nanjing 210008, China; xiongxuan@nju.edu.cn; 4 Nanjing Sport Institute, Nanjing 210014, China

**Keywords:** eating behaviors, autism spectrum disorder, intellectual disabilities, exercises, continuous theta burst stimulation

## Abstract

**Background:** Eating behavior problems significantly affect the physical health and quality of life of children with autism spectrum disorder and intellectual disabilities (co-occurring ASD/ID). This study aimed to evaluate the effects of a 12-week Ball Combination Training Program (BCTP), continuous theta burst stimulation (cTBS), and an intervention combining both (in the CIG) on the eating behaviors of children with co-occurring ASD/ID. **Methods:** A total of 48 participants were assigned into one of four groups: the BCTP (*n* = 13), cTBS (*n* = 12), the CIG (*n* = 11), and a control group (*n* = 12). The intervention groups received their respective treatments in addition to the routine institutional rehabilitation, whereas the control group only received the standard institutional rehabilitation. The intervention outcomes were assessed using the parent-reported Children’s Eating Behavior Questionnaire (CEBQ). **Results:** The results indicated that all three intervention methods led to improvements in their eating behavior after 12 weeks. Specifically, the BCTP group and the CIG demonstrated significantly reduced Food Fussiness behavior, while the children’s Enjoyment of Food was markedly enhanced in the cTBS group and the CIG. Furthermore, the CIG experienced a particularly notable effect in terms of the improvement in the Satiety Responsiveness dimension of their eating behavior. Among the three approaches, the CIG demonstrated a clear advantage over the single interventions in terms of both the breadth and magnitude of its improvements. **Conclusions:** This study confirmed the effectiveness of these three intervention strategies in addressing dietary behavior problems among children with co-occurring ASD/ID. Future research should focus on exploring the combined intervention approach further, particularly its potential synergy, while delving deeper into the neural mechanisms underlying these behavioral improvements.

## 1. Introduction

Autism spectrum disorder (ASD) is a complex neurodevelopmental disorder with a rising prevalence. Its primary characteristics include social communication deficits and restricted repetitive patterns of behavior or interests. Notably, about 70% of children with ASD also present with intellectual disabilities (co-occurring ASD/ID) [1], a group that frequently encounters more severe eating disorders [2]. Research shows that the prevalence of feeding difficulties in children with ASD ranges from 50% to 90% [3], while the occurrence of eating disorders lies between 46% and 89%, which is significantly higher than that in typically developing children [4]. These disordered eating behaviors are closely linked to cognitive and sensory functioning issues within the brain [5]. Consequently, eating behavior problems can lead to malnutrition, obesity, underweight, and even cognitive decline in children with co-occurring ASD/ID [6]. Furthermore, these issues can negatively affect families [7]. Therefore, addressing the eating behavior problems in this specific population of children with co-occurring ASD/ID is crucial.

Exercise is one of the most promising methods for improving eating behavior, especially within the ASD population. Studies by Nabors et al. and Yazar and Alp have shown that both walking and personalized exercise programs can effectively improve eating behaviors in individuals with ASD [8,9]. The potential reason behind these improvements is that exercise enhances prefrontal functions such as cognition and sensory processing, which in turn alleviates the eating disorders associated with ASD. Research suggests that complex ball sports involving multiple limbs may offer greater benefits in terms of cognitive functioning improvements compared to those with simpler aerobic exercises like walking, jogging, or cycling [10]. Therefore, interventions focusing on highly interactive and cognitively engaging ball sports hold significant promise for enhancing dietary behaviors in individuals with ASD. While previous studies have demonstrated the effectiveness of ball sports in improving core symptoms [11], motor impairments [12], and sleep disorders [13] in children with ASD, their impact on eating disorders remains unproven and warrants further investigation. Moreover, it is important to acknowledge the limitations of exercise as an external behavioral intervention, particularly its inability to directly target the prefrontal brain regions associated with eating disorders in ASD.

Repetitive transcranial magnetic stimulation (rTMS) offers promising potential for treating the eating disorders associated with ASD because of its ability to non-invasively and painlessly enhance the function of specific brain regions using magnetic signals [14]. A key area of focus is the dorsolateral prefrontal cortex (DLPFC), a critical brain region involved in decision-making and emotional regulation which significantly impacts eating behavior [15]. Studies have shown that stimulating the DLPFC with either high-frequency or low-frequency rTMS can effectively alter eating behaviors [16,17,18]. Despite its effectiveness, a significant limitation of rTMS is the extended duration required for this intervention. As an alternative, continuous theta burst stimulation (cTBS), a variant of rTMS, can achieve long-term inhibition with brief, low-intensity pulse stimulation [19], offering comparable effects to those of rTMS in a shorter timeframe, making it more suitable for children with ASD. The current research indicates that cTBS can effectively regulate eating behaviors across various groups [20,21]. Nonetheless, the impact of cTBS on eating behavior in children with ASD, particularly those with co-occurring ASD/ID, remains unexplored, warranting further investigation and the validation of intervention outcomes.

Exercise interventions and transcranial magnetic stimulation each have distinct benefits: the former enhances brain cognition and other functions by involving exercise in complex environments, while the latter targets the frontal areas to improve eating behavior disorders. These two approaches can effectively complement each other. In recent years, combined interventions using the two have shown effectiveness in treating conditions such as depression [22], Alzheimer’s disease [23], and Parkinson’s disease [24]. Many studies have found that combined interventions not only prove effective but often yield synergistic effects superior to those of single interventions [25]. The benefits of this synergistic effect are evident in both scope and magnitude, likely due to the complementary nature of the two strategies. Although BCTPs and cTBS are promising interventions and have demonstrated synergy beyond single interventions in addressing sleep issues in children with ASD [13], there has been no research on their combined use (i.e., in a CIG) for eating-related problems, nor any studies targeting the co-occurring ASD/ID population. Therefore, further research is necessary to establish the effectiveness of their combined use and to determine whether a combined intervention is superior to single interventions.

In summary, BCTPs and cTBS both show promising potential in improving eating behaviors. Meanwhile, CIG interventions have proven effective for other issues in children with ASD, and they also hold significant prospects for addressing eating problems. However, the efficacy of these three types of interventions in children with co-occurring ASD/ID remains uncertain. Although combining exercise with transcranial magnetic interventions often yields better results than those of single interventions, these outcomes require further validation. Therefore, the primary objectives of this study are as follows:

O1: To investigate the impact of a BCTP on the eating behaviors of children with co-occurring ASD/ID;

O2: To explore the effects of cTBS on the eating behaviors of children with co-occurring ASD/ID;

O3: To examine the influence of a combined intervention on the eating behaviors of children with co-occurring ASD/ID;

O4: To compare the effectiveness of combined interventions with that of single interventions.

This study aims to explore the potential of BCTP, cTBS, and CIG interventions to address eating behavior problems in children with co-occurring ASD/ID. Additionally, it seeks to determine whether combined interventions are more effective than single interventions. This research will provide guidance for improving the eating behaviors of children with autism and intellectual disabilities, alleviating family burdens, and enhancing quality of life.

## 2. Materials and Methods

### 2.1. The Study Design

This study employed a 4 (BCTP, cTBS, CIG, CG) × 2 (pre-test, post-test) experimental design, with approval from the Ethics and Human Protection Committee of the Affiliated Hospital of Yangzhou University (Approval No. YXYLL-2023-147). All of the study procedures adhered to the latest version of the Declaration of Helsinki. Prior to this study, the research goals were thoroughly explained to the parents of the participating children, and written informed consent was obtained from all of the participants.

### 2.2. The Participants

This study, a four-arm controlled trial, was conducted from June to September 2023 at the Yangzhou Eagle Child Development Centre. A total of 77 children with ASD recruited from this institution met the DSM-5 diagnostic criteria. The inclusion criteria specified children aged 4–12, of Han ethnicity, with a clinical diagnosis of ASD, and an IQ below 70 as measured by the Wechsler Intelligence Scale who provided informed consent, along with being willing to participate. The exclusion criteria encompassed severe head trauma or metal foreign objects in the skull; coexisting neurological or psychiatric disorders; implanted devices such as pacemakers, implantable defibrillators, or neurostimulators; medication use affecting the central nervous system; physical disabilities; visual or auditory impairments; and prior transcranial magnetic stimulation treatment.

**Figure 1:**
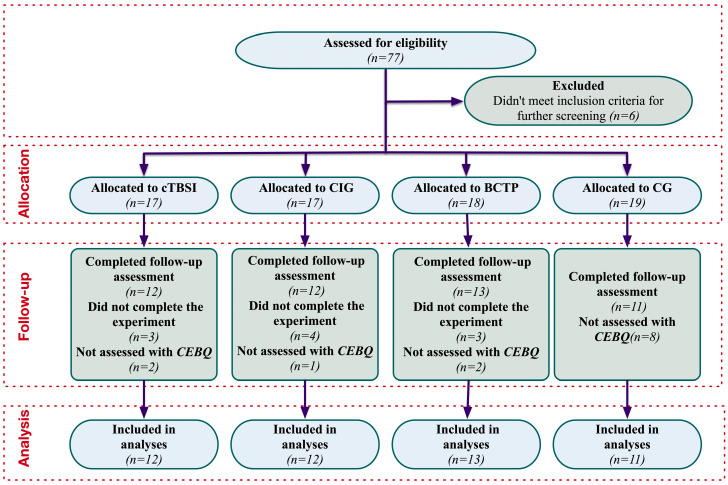
Study flowchart.

The participants were categorized into four groups considering their age, their gender, the severity of their autism symptoms (as classified by the institution according to function and symptoms), and their enrollment date. The groups included a Ball Combination Training Program (BCTP) group with 17 children, continuous theta burst stimulation (cTBS) in 17 children, a combined intervention group (CIG) of 18 children, and a control group (CG) with 19 children. This grouping strategy ensured that baseline functional levels were balanced across the groups while also accommodating the institution’s teaching schedule, and the details of the subject screening process are illustrated in Figure 1.

### 2.3. The Ball Combination Training Program

The BCTP intervention aligned with programs from previous studies [12,13] and added mini-football interventions to these existing mini-basketball interventions [11,26]. Conducted daily from 3 PM to 4 PM over 12 weeks, the program holds sessions five times per week, with each session lasting 35 min. Designed specifically for children with ASD, the BCTP uses smaller, lighter basketballs that are suitable for both football and basketball, accommodating the children’s shorter stature, smaller hands, and lower strength. The combination of mini-football and mini-basketball interventions not only diversifies the intervention content but also provides a comprehensive physical stimulus by engaging both the upper and lower limbs. Implemented by trained professionals with a bachelor’s degree in sports science and teaching credentials, the BCTP ensures a focused setting by limiting the number of students to fewer than ten per session and requiring at least two teachers, considering the special needs of children with ASD and intellectual disabilities. Guardians are encouraged to accompany their children during the interventions. For further details on the course objectives, schedules, and implementation methods, please refer to the Supplementary Materials.

### 2.4. The Continuous Theta Burst Stimulation Intervention

The cTBS protocol was administered using the Rapid2 transcranial magnetic stimulation (TMS) device (Magstim Inc., Whitland, UK) equipped with a figure-of-eight coil. The participants in the cTBS group and the CIG received pattern stimulation via cTBS three times daily, with a 15 min interval between sessions, from Monday to Friday, totaling 12 weeks of intervention. The stimulation site was the left DLPFC for the first six weeks and the right DLPFC for the latter six weeks. The cTBS sessions were conducted from 9 to 11 AM. The resting motor threshold was determined by measuring the muscle response of the adductor muscles and set at 80%. According to the current guidelines, each cTBS stimulation consisted of three pulse trains at a frequency of 50 Hz (5 Hz every 200 ms), with each intervention lasting 40.02 s and comprising 600 pulses. The cTBS interventions were administered by trained and qualified rehabilitation therapists. For more details, see the Supplementary Materials.

### 2.5. The Procedure

**Figure 2:**
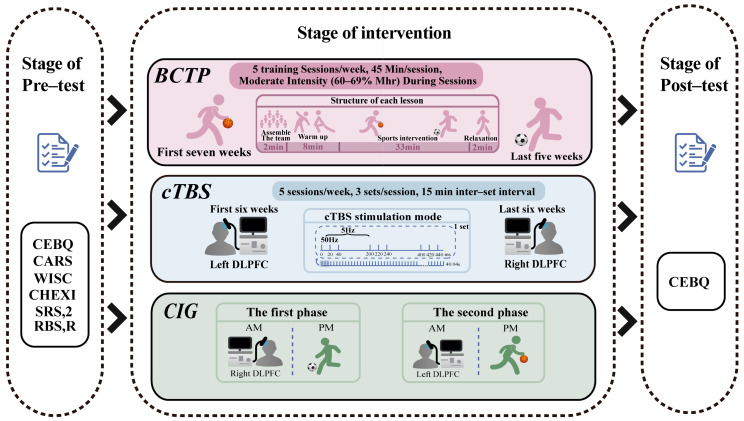
A schematic representation of the study protocol.

The overall experiment was divided into three phases: pre-test, intervention, and post-test (see Figure 2). The pre-test was conducted one week before the intervention to establish a baseline, while the post-test was conducted immediately afterwards to assess its immediate effects. All of the participants engaged in daily rehabilitation training at the institution, utilizing methods such as an Applied Behavior Analysis (ABA), Floor Time, a Relationship Development Intervention (RDI), and TEACCH. In addition to the routine rehabilitation, the cTBS group received cTBS stimulation, and the BCTP group underwent the BCTP intervention, while the CG only followed the routine training. The CIG participated in both the cTBS and BCTP interventions; they received cTBS stimulation in the morning and the BCTP intervention in the afternoon, ensuring a structured daily schedule. This design ensured that all of the interventions were consistent with each other, maintaining the integrity of the experimental conditions.

### 2.6. The Control Variable

In the preliminary assessment, demographic data (age, gender) for the participants were collected, as detailed in Table 1. The Childhood Autism Rating Scale (CARS) and the Wechsler Intelligence Scale for Children, Fourth Edition (WISC-IV), were utilized to assess the presence and severity of autism, as well as the IQ levels of the participants. Recognizing the influence of executive function [27], sleep patterns [28], repetitive behaviors [29], social interactions [30], and dietary habits on autism, as suggested by previous studies, comprehensive assessments were conducted. These included the Child Executive Functioning Inventory (CHEXI), the Children’s Sleep Habits Questionnaire (CSHQ), the Repetitive Behavior Scale, Revised (RBS-R), and the Social Responsiveness Scale, Second Edition (SRS-2), to control for these confounding variables. The CARS assessment was administered by a hospital-based medical professional, the WISC-IV was applied by a qualified experimenter, and the SRS-2, RBS-R, CSHQ, and CHEXI assessments were completed by the participants’ parents.

### 2.7. The Outcome Measures

The results of this study are reported using the Children’s Eating Behavior Questionnaire (CEBQ), which has been culturally adapted from its original English version and validated for use in China with good reliability and validity (Cronbach’s α > 0.7). The CEBQ comprises 35 items distributed across eight dimensions, with each assessing different aspects of children’s eating behaviors. These dimensions are Food Responsiveness (FR), Enjoyment of Food (EF), Emotional Overeating (EOE), Desire to Drink (DD), Satiety Responsiveness (SR), Slowness in Eating (SE), Emotional Undereating (EUE), and Food Fussiness (FF). Each dimension contains 3–6 items, scored on a scale from 1 (“never”) to 5 (“always”). Notably, the items Q3, Q4, Q10, Q16, and Q32 use reverse scoring. The overall score for each dimension is calculated as the average of its items, with higher scores indicating more frequent behaviors in that dimension.

### 2.8. The Statistical Analysis

In this study, the statistical tests were two-tailed, with a significance threshold set at 0.05. We conducted our analyses using SPSS 29.0.2 and R version 4.3.1, incorporating all of the data collected after the baseline. The baseline characteristics of the sample included frequencies, mean values, and 95% confidence intervals. For the analysis of continuous variables, those that followed a normal distribution were subjected to a one-way ANOVA, whereas those that did not follow a normal distribution were analyzed using non-parametric tests. Categorical variables were examined using the chi-square test.

The effectiveness of the three intervention methods in improving the eating behavior of the children with ASD was assessed using a mixed-effects model for repeated measures (MMRM). All of the dimensions were corrected for multiple comparisons using Bonferroni correction. The model accounted for time, group, and their interaction as fixed effects and included random intercepts of the subjects as random effects to address the baseline differences among the participants. After the initial model setup, BMI was incorporated as an additional fixed effect to control for confounding variables. The adequacy of the model, including BMI, was evaluated using a likelihood ratio test (LRT). The results were presented as least squares means (LS means) and 95% confidence intervals (CIs).

In this study, we compared the effectiveness of the three intervention methods by calculating the effect sizes of each intervention relative to that for the control group. These calculations were based on the estimated marginal means derived from the MMRM. The effect sizes were quantified using Cohen’s d-values, and the results were reported alongside the 95.

## 3. Results

### 3.1. Demographic Characteristics

The demographic results at the baseline are presented in the Table 1. The subjects had an average CARS score of 36.54, indicative of severe autism symptoms (clinically diagnosed as ASD), and an average IQ of 48.10, with all of the subjects having an IQ below 70, confirming intellectual disability. In terms of ASD’s core symptoms, no significant differences were observed in their repetitive stereotyped behaviors and social impairments (Ps > 0.05). Similarly, no significant differences were found across various dimensions of their baseline dietary habits (Ps > 0.05).

### 3.2. The Effect of the Three Intervention Methods on Eating Behavior

**Figure 3:**
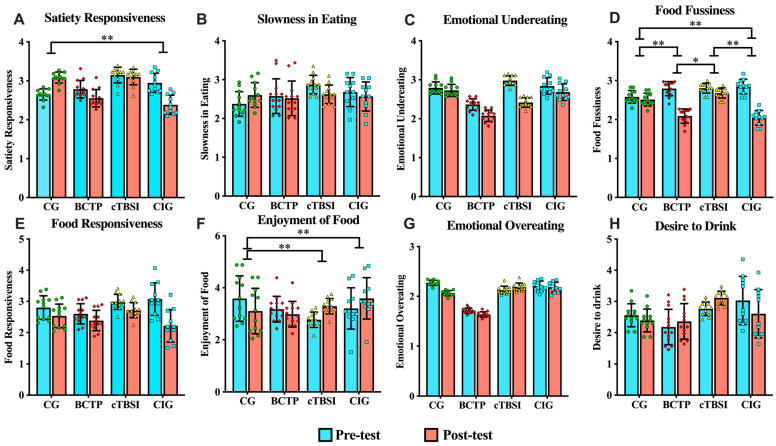
Changes in eating behavior before and after the interventions across different groups. Time×group interaction effect plots for each dimension of the CEBQ scale. Significance levels are marked as * for 0.01 < *p* ≤ 0.05, ** for 0.001 < *p* ≤ 0.01.

The results in terms of the effect of the time × group interaction of the three intervention methods on the dietary behaviors of the children with co-occurring ASD/ID, as analyzed using the MMRM, are depicted in Figure 3. Both the BCTP and the combined intervention were effective in improving the children’s picky eating behaviors, while cTBS and the combined intervention positively influenced the children’s enjoyment of food. Additionally, the CIG experienced a significant impact on the Satiety Responsiveness dimension of its eating behavior. However, none of the intervention methods demonstrated significant effects on the other dimensions of eating behavior.

#### 3.2.1. The Effect of the BCTP on the Children’s Eating Behavior

Compared to the control group, the BCTP intervention significantly improved the Food Fussiness dimension in the children with co-occurring ASD/ID, with an LSM.D of −0.64 (95% CI: −1.23 to −0.04, *p* = 0.010). Additionally, the improvement effect of the BCTP was notably better than that of cTBS, with an LSM.D of −0.58 (95% CI: −1.17 to 0.01, *p* = 0.015). In terms of Satiety Responsiveness, the improvement effect of the BCTP was near-significant compared to this dimension in the control group, with an LSM.D of −0.664 (95% CI: −1.47 to 0.14, *p* = 0.081). However, the intervention effects of the BCTP on the other dimensions of eating behavior were not significant.

#### 3.2.2. The Effect of cTBS on the Children’s Eating Behavior

The cTBS intervention significantly improved the Enjoyment of Food dimension in the children with co-occurring ASD/ID, with an LSM.D of 1.00 (95% CI: 0.1258 to 1.874, *p* = 0.017). However, this intervention did not significantly improve their other eating behaviors.

#### 3.2.3. The Effect of the Combined Intervention on the Children’s Eating Behavior

The CIG of children with ASD had significantly improved Satiety Responsiveness (LSM.D = −0.99; 95% CI: −1.83 to −0.16; *p* = 0.012), Food Fussiness (LSM.D = −0.73; 95% CI: −1.35 to −0.12; *p* = 0.010), and Enjoyment of Food (LSM.D = 0.87; 95% CI: −0.03 to 1.76; *p* = 0.031). In terms of Food Fussiness, the improvement effect in the CIG was significantly better than that with cTBS, with an LSM.D of −0.68 (95% CI: −1.30 to −0.06, *p* = 0.010). However, the CIG did not show significant improvement effects in the other dimensions of its eating behavior.

### 3.3. Comparing the Effects of the Three Interventions

**Figure 4:**
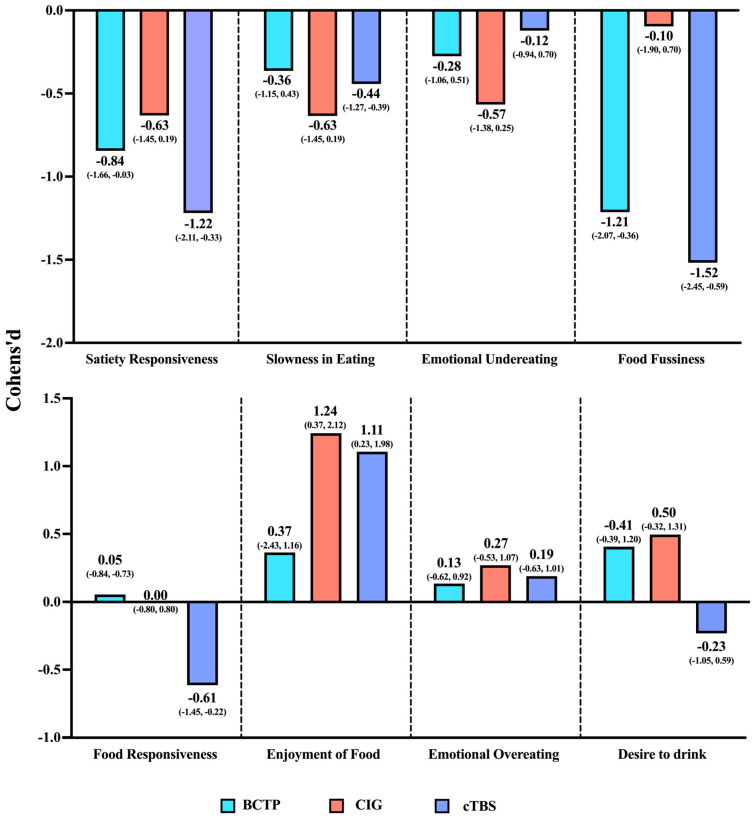
Results of effect sizes for the three interventions compared to the control group. Note: The bar graph presents the Cohen’s d effect sizes and their 95% CIs for the differences between the post-intervention and pre-intervention measurements across various dimensions compared to those in the control group.

Figure 4 presents the comparative effect sizes of three intervention modalities relative to CG. For eating behavior issues in children with co-occurring ASD/ID, the combined intervention demonstrated superiority in both the breadth and magnitude of its effects over these for single interventions.

In the dimensions of Satiety Responsiveness and Food Fussiness where significant improvements were observed, the CIG intervention was more effective than both cTBS and the BCTP. In the Enjoyment of Food dimension, although the effect size of the cTBS intervention was larger, the combined intervention still maintained a substantial effect size. In the dimensions where the intervention effects were not significant, all three interventions showed a trend towards improvements in Slowness in Eating and Emotional Undereating.

Specifically, in the dimension of Satiety Responsiveness, the improvement effect in the CIG (Cohen’s d = −1.22, 95% CI: −2.11 to −0.33) was significantly better than that of the BCTP (Cohen’s d = −0.84, 95% CI: −1.66 to −0.03) and cTBS (Cohen’s d = −0.63, 95% CI: −1.45 to 0.19). In the dimension of Food Fussiness, although both the CIG and the BCTP group showed significant improvements, the effect in the CIG (Cohen’s d = −1.52, 95% CI: −2.45 to −0.59) was notably better than that in the BCTP group (Cohen’s d = −1.21, 95% CI: −2.07 to −0.36). In the dimension of Enjoyment of Food, the intervention effect in the CIG (Cohen’s d = 1.11, 95% CI: 0.23 to 1.98) was inferior to that under cTBS (Cohen’s d = 1.24, 95% CI: 0.37 to 2.12).

## 4. Discussion

This study explored the impact of 12-week BCTP, cTBS, and combined interventions on children with co-occurring ASD/ID, assessing whether the combined intervention surpassed the efficacy of that of the individual treatments. The findings reveal that the BCTP significantly reduces Food Fussiness in children with ASD/ID, cTBS markedly enhances Enjoyment of Food, and the combined intervention notably improves Satiety Responsiveness, Food Fussiness, and Enjoyment of Food. Notably, the combined intervention demonstrates a synergistic effect that is more pronounced than that of the individual interventions.

This study explored the effects of the BCTP on the eating behaviors of children with co-occurring ASD/ID, revealing that the BCTP significantly improved Food Fussiness, though it had no significant impact on the other dimensions of eating behavior. Selective/avoidant eating, a severe issue in children with ASD [31], has been linked to physical activity levels, which influence food choice behaviors [32]. Furthermore, exercise interventions have been shown to effectively alleviate picky eating [33]. The potential mechanisms behind the BCTP’s effectiveness include its impact on repetitive behaviors and restricted interests [29], as are common in ASD, which are mitigated by activities like ball sports that alter brain network connections [34], thus reducing Food Fussiness. Additionally, sensory sensitivity, a significant factor in Food Fussiness [35,36], can be addressed through motor activities that engage multiple sensory systems, effectively reducing symptoms including selective eating [37]. Exercise also enhances fine motor skills, improving the oral processing of food in children with ASD [38]. Despite the BCTP’s significant improvement in Food Fussiness, its effects on the other eating behaviors were not significant, possibly due to the short duration of the intervention. However, many dimensions exhibited trends of improvement, indicating the need for further research to fully understand the BCTP’s impact on eating behaviors and its underlying physiological mechanisms.

Additionally, we explored the effects of cTBS on the eating behaviors of children with co-occurring ASD/ID. The findings revealed that cTBS significantly enhanced Enjoyment of Food among these children, although it had no notable impact on the other aspects of their eating behaviors. It is well documented that children with ASD often exhibit low interest in food, which can lead to malnutrition and subsequently to either underweight or overweight conditions [39]. Previous studies have demonstrated that cTBS can increase food cravings in children with ASD [40]. This enhancement in Enjoyment of Food may stem from cTBS’s influence on cognitive functions related to inhibition, which reduces control over preferred foods and heightens eating satisfaction. Moreover, the DLPFC is integral to regulating eating behaviors [41], and cTBS may improve its function by enhancing inhibitory control and correcting the imbalance in the excitatory/inhibitory (E/I) ratio caused by overexcitation of the DLPFC cortex in ASD. During the 12-week intervention of this study, cTBS specifically improved the Enjoyment of Food dimension. We hypothesize that cTBS’s effectiveness in this area may be due to its particular efficacy in enhancing tendency-driven eating behaviors in ASD. Therefore, further research is needed to explore the neural mechanisms through which cTBS improves Enjoyment of Food and its potential effects on avoidant eating behaviors.

Finally, we investigated the impact of a controlled intervention on the eating behaviors of children with co-occurring ASD/ID, comparing the efficacy of combined interventions to that of single interventions. The results indicated that the CIG had significantly enhanced Satiety Responsiveness, Food Fussiness, and Enjoyment of Food. In the areas where the single interventions had been effective, the results from the CIG not only matched but exceeded their effectiveness, demonstrating superior synergistic effects. Although prior research on combined interventions for eating behaviors is lacking, similar synergistic effects have been observed in studies involving exercise and cTBS for sleep disorders in children with ASD [13]. The synergistic effects in the CIG may be attributed to several mechanisms. Firstly, the BCTP broadly stimulates the body, affecting a wide range of stimuli, while cTBS targets specific brain areas related to eating, providing precise stimulation. This complementary approach allows for a more comprehensive impact on eating behaviors. Additionally, the BCTP and cTBS each improve different aspects of eating behaviors; the BCTP addresses the changes caused by the repetitive and stereotypical symptoms of ASD, and cTBS enhances the function of the DLPFC, a brain area crucial for eating regulation. Consequently, the BCTP is particularly effective in mitigating picky and avoidant eating behaviors, whereas cTBS significantly boosts Enjoyment of Food. Therefore, the combination of cTBS and the BCTP not only ameliorates the eating behaviors of children with co-occurring ASD/ID but also achieves greater synergistic effects than those of single interventions, both in their scope and intensity. However, the scarcity of research, particularly on combined interventions in the dietary aspects of ASD, necessitates further studies to elucidate the neural mechanisms and intervention strategies underlying these synergistic effects.

## 5. Limitations

This study investigated combined interventions in the eating behaviors of children with co-occurring ASD/ID, revealing that cTBS, the BCTP, and a combined intervention enhanced specific dimensions of their eating behaviors. However, it is crucial to acknowledge that not all of the dimensions showed improvement. This lack of improvement may have stemmed from limitations in the intervention methods or from several research shortcomings: Firstly, the intervention techniques and stimulation parameters for the BCTP and cTBS require further refinement. Secondly, the CEBQ scale used for the evaluation is subjective and may not have accurately reflected the actual eating behaviors of the participants. In addition, this study did not sufficiently consider the influence of family dynamics on eating behaviors. Thirdly, the overall duration of the intervention might have been too short to influence certain eating behaviors effectively. Finally, the BCTP requires extensive educational resources, resulting in a small sample size and challenges in maintaining a strict randomization process. At the same time, it is difficult to consider the impact of gender differences on the results, which may compromise the stability of the findings. Future studies should consider extending the duration of the intervention, conducting thorough follow-ups, adhering strictly to the randomization process, and increasing the sample size to enhance the reliability of the results. At the same time, attention will be given to the influence of families on the eating behaviors of children with ASD, as well as improvements in children’s food complaint behaviors.

## 6. Conclusions

This study aimed to investigate whether 12-week cTBS, BCTP, and CIG interventions could improve the eating behaviors of children with co-occurring ASD/ID and to compare whether combined interventions could produce better synergistic effects than individual interventions alone. The results indicated that cTBS significantly enhanced Enjoyment of Food, the BCTP notably improved Food Fussiness, and the combined intervention positively affected Enjoyment of Food, Food Fussiness, and Satiety Responsiveness, thereby demonstrating superior synergistic effects compared to those of the single interventions. Moreover, this study confirmed the feasibility of integrating physical and transcranial magnetic interventions. Further research is required to elucidate the mechanisms through which these three interventions ameliorate the eating behaviors of children with co-occurring ASD/ID.

## Figures and Tables

**Table 1:** Baseline characteristics of participants.

Variable	Mean/Proportion (95% CI)					
	Entire Sample	CG	cTBS	BCTP	CIG	*p*
	(N = 48)	(N = 12)	(N = 12)	(N = 13)	(N = 11)	Value
Age (year), mean	7.27 (6.56, 7.99)	8.83 (7.06, 10.61)	6.75 (5.48, 8.02)	6.46 (5.05, 7.87)	7.09 (5.61, 8.58)	0.07
Sex (%)						0.69 ^a^
Male	73.33	83.4	66.67	76.93	63.64	
Female	27.08	16.6	33.33	23.07	36.36	
IQ score, mean ^b^	48.10 (45.69, 50.52)	46.00 (39.80, 52.19)	45.42 (42.09, 48.74)	49.15 (43.75, 54.56)	52.09 (46.79, 57.39)	0.19
Autism Rating Scale score, mean ^c^	36.54 (35.03, 38.05)	36.33 (33.02, 39.64)	38.00 (35.50, 40.50)	35.69 (31.67, 39.72)	36.18 (32.96, 39.40)	0.73
Repetitive Behavior Scale score, mean ^d^	27.26 (23.05, 31.46)	27.50 (20.10, 34.90)	32.75 (21.41, 44.09)	25.33 (18.41, 32.26)	23.09 (12.37, 33.81)	0.42
Social impairment score, mean ^e^	95.90 (90.14, 101.65)	92.58 (79.42, 105.74)	95.83 (84.00, 107.67)	97.77 (85.60, 109.94)	97.36 (82.52, 112.21)	0.92
Executive function ^f^						
Working memory	47.40 (45.28, 49.51)	47.33 (42.03, 52.64)	47.92 (43.85, 57.98)	47.55 (41.82, 53.27)	46.85 (42.80, 50.89)	0.99
Inhibition	38.48 (36.18, 40.78)	38.08 (31.00, 45.17)	38.50 (35.19, 41.81)	39.38 (35.39, 43.37)	37.82 (32.06, 43.58)	0.97
Eating behavior ^g^						
Satiety Responsiveness (SR)	2.88 (2.72, 3.04)	2.65 (2.37, 2.93))	3.15 (2.79, 3.51)	2.78 (2.49, 3.08)	2.95 (2.54, 3.35)	0.13
Slowness in Eating (SE)	2.63 (2.45, 2.80)	2.38 (2.03, 2.72)	2.88 (2.63, 3.12)	2.58 (2.13, 3.02)	2.68 (2.23, 3.13)	0.25
Emotional Undereating (EUE)	2.73 (2.53, 2.94)	2.79 (2.29, 3.28)	2.98 (2.50, 3.46)	2.37 (2.03, 2.70)	2.84 (2.38, 3.30)	0.16
Food Fussiness (FF)	2.76 (2.61, 2.90)	2.58 (2.32, 2.85)	2.81 (2.49, 3.12)	2.79 (2.47, 3.12)	2.85 (2.47, 3.33)	0.59
Food Responsiveness (FR)	2.85 (2.61, 3.09)	2.80 (2.14, 3.46)	2.98 (2.60, 3.36)	2.60 (2.29, 2.91)	3.07 (2.34, 3.80)	0.52
Enjoyment of Food (EF)	3.18 (2.92, 3.44)	3.58 (2.81, 4.35)	2.77 (2.50, 3.04)	3.17 (2.79, 3.56)	3.20 (2.51, 3.90)	0.13
Emotional Overeating (EOE)	2.07 (1.87, 2.27)	2.27 (1.82, 2.72)	2.13 (1.62, 2.63)	1.71 (1.46, 1.96)	2.20 (1.70, 2.71)	0.18
Desire to Drink (DD)	2.61 (2.34, 2.89)	2.56 (2.16, 2.95)	2.76 (2.34, 3.18)	2.18 (1.62, 2.74)	3.03 (2.12, 3.94)	0.16

^a^ Calculated using the chi-square test. ^b^ Estimated from the Wechsler Intelligence Scale for Children, Fourth Edition (WISC-IV). ^c^ Estimated from the Childhood Autism Rating Scale (CARS). ^d^ Estimated from the Repetitive Behavior Scale, revised (RBS,R). ^e^ Estimated from the Social Responsiveness Scale, Second Edition (SRS,2). ^f^ Estimated from the Child Executive Functioning Inventory (CHEXI). ^g^ Estimated from the Children’s Eating Behavior Questionnaire (CEBQ).

## Data Availability

The data are available: the datasets generated during and/or analyzed during the current study are available from the corresponding author on reasonable request.

## Supplementary Materials

The following supporting information can be downloaded at https://www.mdpi.com/article/10.3390/nu17091446/s1.
