# Supplementary material for: The Effects of a Ball Combination Training Program Combined with a Continuous Theta Burst Stimulation Intervention on Eating Behaviors in Autistic Children with Accompanying Intellectual Disabilities: A Preliminary Study
Source: Nutrients. 2025 Apr 25;17(9):1446. doi: 10.3390/nu17091446 (PMC12073229; doi:10.3390/nu17091446)
Supplement: Supplementary file 1 [file nutrients-17-01446-s001.zip › nutrients-3559486-supplementary.pdf]

Intervention Program: The Effects of a Ball Combination Training Program Combined with a Continuous Theta Burst Stimulation Intervention on Eating Behaviors in Autistic Children with Accompanying In-tellectual Disabilities: A Preliminary Study

Funding: This work was supported by the National Social Science Foundation of China [grant numbers 23ATY008] and The Fok Ying Tong Education Foundation [grant number 141113]

Protocol:

Background:

Dietary behavior problems significantly impact the physical health and quality of life of children with Autism Spectrum Disorder (ASD). A high percentage, ranging from 50% to 90%[1], of children with ASD face eating difficulties, with the prevalence of eating disorders between 46% and 89%, far exceeding that in neurotypical children[2]. These issues can lead to severe outcomes such as malnutrition and life-threatening conditions, with affected children often being either overweight or underweight[3]. Therefore, early intervention in addressing these dietary behaviors is essential for enhancing cognitive development and promoting overall physical health in children with ASD.

Currently, no specific medications exist for ASD, but evidence supports physical activity as an effective strategy for dietary improvement[4]. Ball sports, in particular, have been proven to benefit social impairments and repetitive behaviors in children with ASD[5,6]. Due to their ease of implementation, frequent interaction, engaging nature, and moderate intensity, combined ball sports offer unique advantages in interventions for these children. However, it is important to note that combined ball sports, while beneficial, cannot target specific brain regions directly.

Research has linked the dietary behavior abnormalities in children with ASD to the cognitive control functions of the dorsolateral prefrontal cortex (DLPFC) [7]. The DLPFC serves as a central modulator of cognitive functions, and imbalances in its excitatory-inhibitory ratio due to ASD development can be addressed by continuous Theta Burst Stimulation (cTBS). cTBS can stimulate the DLPFC, promoting changes in the cortical network and subcortical plasticity, thus enhancing its function and improving the dietary behaviors of children with ASD[8].

However, the effects of cTBS, combined ball sports, or their joint interventions on the dietary behaviors of children with ASD have not yet been proven., and the superiority of combined interventions over single interventions requires further investigation. We hypothesize that all three interventions can improve these behaviors, with combined interventions potentially offering greater benefits. This study explores the advantages of a combined intervention program involving mini-basketball and mini-soccer, alongside cTBS interventions targeting both sides of the DLPFC, in improving the dietary behaviors of

children with co-occurring ASD/ID.

Study design

This study is a four-arm controlled experiment conducted from June to September 2023 at the Yangzhou Eagle Child Development Centre in China. Participants were divided into four groups based on their age, gender, severity of autism symptoms (children were classified by the institution based on functionality and symptoms), and enrollment time. The groups included the Ball Combination Training Program (BCTP, 17 children), Continuous Theta Burst Stimulation (cTBS, 17 children), Combined Intervention Group (CIG, 18 children), and Control Group (CG, 19 children). This design ensures that the baseline functional levels of the participants in each group are balanced while accommodating the teaching arrangements of the institution.

Participants:

The subjects, aged between 4 and 12 years old, were clinically diagnosed with ASD according to the DSM-5 criteria. Prior to the experiment, the severity of autism in each child was reassessed using the Childhood Autism Rating Scale (CARS), and their intelligence was evaluated with the fourth edition of the Wechsler Intelligence Scale for Children (WISC-IV). This age range was specifically chosen because it represents a critical period for the children's growth and development.

Inclusion Criteria: (1) Aged 4-12 years. (2) Of Han ethnicity. (3) Clinically diagnosed with ASD. (4)Intelligence quotient (IQ) measured below 70 using the Wechsler Intelligence Scale. (5) Provided informed consent and voluntarily participated.

Exclusion Criteria: (1) Severe head trauma or intracranial metal objects. (2) Accompanying neurological or psychiatric disorders. (3) Presence of a cardiac pacemaker. (4)Implanted devices such as defibrillators or neurostimulators. (5) Currently taking medications that affect the central nervous system. (6) Physical disabilities. (7) Visual or auditory impairments. (8)Previous treatment with transcranial magnetic stimulation. (9)Previous participation in ball sports interventions.

Ethics:

The study follows the Helsinki Declaration. The research protocol has been reviewed and approved by the Ethics Committee of the Yangzhou University Medical School [approval number: YXYLL-2023-147]

Ball Combination Training Program (BCTP):

Spanning 12 weeks, the BCTP intervention was delivered once daily from Monday to

Friday, resulting in a total of five weekly sessions. Table details the weekly intervention plan for BCTP. Intervention timeframes and venues remained uniform throughout the program. The curriculum's complexity was amplified incrementally, progressing through three stages: acclimatization, foundation, and advancement.

The primary goals of these respective stages are outlined as follows:

Phase Ⅰ (Adaptation Phase):The initial phase, termed the adaptation phase, is designed to facilitate the children's acclimatization to the structured course and environment. This phase emphasizes the cultivation of social etiquette and behavioral norms. Subsequently, the program enhances children's interest in physical activities through engaging games that also promote physical fitness.

Phase II (Foundation Phase):Following the adaptation phase is the basic phase. This stage concentrates on assisting children to learn and perfect fundamental basketball and soccer skills. In tandem with this, the program aims to augment the acquisition of complex motor skills and address prevalent behavioral concerns or detrimental habits commonly observed in children with ASD during the course.

Phase III (Advanced Phase):The third phase, known as the enhancement phase, introduces various scenario-based activities. These activities are crafted to encourage children to flexibly apply motor skills across diverse environments, enhance their awareness of cooperation, and improve social skills, thereby activating their social capabilities.

Table : Ball Combination Training Program process

		BCTP Training Drills			
		**Assemble the team (2min)**	**Warm up (8min)**	**Sports intervation (33min)**	**Relaxation (2min)**
**Phase I** (Adaptation Phase)	week 1	Assemble the team	Warm up	Mini-basketball tactile desensitization, Mini-basketball visual desensitization, Physical fitness exercises (return running with the ball, etc.), Sports games (high-five passing games, etc.)	Relaxation
**Phase II** (Foundation Phase)	week 2	Assemble the team	Warm up	Basic mini-basketball skills learning (such as passing, dribbling, shooting, etc.), Physical fitness exercises, Sports games (jumping jacks + two-handed dribbling, seeing who can react faster, etc.)	Relaxation
	week 3	Assemble the team	Warm up	Basic mini-basketball skills learning (such as passing, dribbling, shooting, etc.), physical fitness exercises, Sports games (jumping jacks + two-handed dribbling, seeing who can react faster, etc.)	Relaxation
	week 4	Assemble the team	Warm up	Basic mini-basketball skills learning (such as passing, dribbling, shooting, etc.), physical fitness exercises, Sports games (jumping jacks + two-handed dribbling, seeing who can react faster, etc.)	Relaxation
	week 5	Assemble the team	Warm up	Basic mini-basketball skills learning (such as passing, dribbling, shooting, etc.), physical fitness exercises, Sports games (jumping jacks + two-handed dribbling, seeing who can react faster, etc.)	Relaxation
	week 6	Assemble the team	Warm up	Basic mini-basketball skills learning (such as passing, dribbling, shooting, etc.), physical fitness exercises, Sports games (jumping jacks + two-handed dribbling, seeing who can react faster, etc.)	Relaxation
	week 7	Assemble the team	Warm up	Basic mini-basketball skills learning (such as passing, dribbling, shooting, etc.), physical fitness exercises, Sports games (jumping jacks + two-handed dribbling, seeing who can react faster, etc.)	Relaxation
	week 8	Assemble the team	Warm up	Basic technical learning of football (such as kicking a spot kick, alternating feet on the ball and dribbling with the inside of the foot, etc.), Physical fitness exercises, Sports games (such as spot dribbling and one-foot dribbling around obstacles, etc.)	Relaxation
	week 9	Assemble the team	Warm up	Basic technical learning of football (such as kicking a spot kick, alternating feet on the ball and dribbling with the inside of the foot, etc.), Physical fitness exercises, Sports games (such as spot dribbling and one-foot dribbling around obstacles, etc.)	Relaxation
	week 10	Assemble the team	Warm up	Integrate 2-9 weeks of mini-basketball and soccer skills learning	Relaxation
**Phase III** (Advanced Phase)	week 11	Assemble the team	Warm up	Combine the basic movement techniques of mini-basketball and football to design group sports games, such as relay throwing and catching, dribbling relay games, small circle shooting and large circle jumping games, etc.	Relaxation
	week 12	Assemble the team	Warm up	Combine the basic movement techniques of mini-basketball and football to design group sports games, such as relay throwing and catching, dribbling relay games, small circle shooting and large circle jumping games, etc.	Relaxation

The BCTP intervention was implemented by academically trained physical education students, all of whom also possessed formal teaching certifications. The structure was one of organized groups, with each encompassing 5 to 10 children diagnosed with autism - a strict limit of 10 precluded any larger class sizes. Every cohort contained a primary instructor tasked with intervention deployment, supplemented by a secondary instructor tasked with the management of classroom dynamics and additional support. Conscious of the unique difficulties experienced by co-occurring ASD/ID children in the context of independent class participation, the initiative encouraged the accompaniment of guardians. Rigorous record keeping was maintained for attendance - chronicled for every individual participant at each class by the instructors. The participatory guidelines stipulated a maximum allowance of a total of 5 missed sessions. Moreover, any participant absent for 3 consecutive sessions would necessitate their exclusion from the study to maintain the consistency and integrity of the intervention.

continuous Theta Burst Stimulation (cTBS)

The cTBS intervention employs the UK-manufactured Rapid2 transcranial magnetic stimulation (TMS) device from Magstim Company, equipped with a figure-eight coil. The overall intervention consists of 180 sessions over 12 weeks, conducted three times per day from Monday to Friday, with each session spaced 15 minutes apart. The stimulation is administered in cTBS mode, using three pulse trains delivered at a frequency of 50 Hz every 200 milliseconds (5 Hz). Each intervention lasts 40.02 seconds, providing a total of 600 pulses across the three daily sessions. The specific stimulation protocol is illustrated in Figure 2 (main text). The cTBS targets the left and right DLPFC, with the right DLPFC being stimulated after the traditional left-side stimulation .

During the intervention, participants wore a positioning cap for accurate localization. The motor cortex (M1) and DLPFC were located using the EEG-based “5 cm rule,” with Cz defined as the intersection of the nasion-inion line and the preauricular line. The left primary M1 was located by moving 5 cm to the left along the preauricular line from Cz, and the left DLPFC in the left hemisphere was defined by moving 5 cm forward from M1. The resting motor threshold (RMT) was measured visually by placing the hand palm up on the knee in a relaxed state, stimulating the corresponding M1 area of the contralateral cortex with a TMS stimulation coil, and evoking motor evoked potentials greater than 50 μV in at least 5 out of 10 consecutive TMS stimulations. The minimum stimulus intensity required to elicit a contractile movement of the finger muscle was measured as a percentage of the minimal intensity of the stimulus.

The stimulus intensity of cTBS was maintained at 80% of the resting motor threshold (RMT). cTBS interventions were delivered by two professionals qualified in transcranial magnetic stimulation. In this study, children were stimulated on a one-to-one basis. During the stimulation process, the experimental protocol was strictly adhered to. In case of a pause

in the experiment, the stimulation was resumed after the next child had finished, and if the child experienced any discomfort, the stimulation was immediately paused, the reason was asked, and the cause was recorded. Attendance was taken before the start of each day's intervention, and parents were asked to communicate with the teacher one day in advance if the child needed to be absent from work. The number of consecutive days of absence from work during the intervention process could not exceed 3, and the cumulative number of days of absence from work could not exceed 7, and the children who did not satisfy the frequency of the interventions would be excluded from the interventions.

Intervention Overall Program

The overall experiment was divided into three phases: pre-test, intervention, and post-test (see figure 2 ,main text). The pre-test was conducted one week before the intervention to establish a baseline, and the post-test was conducted immediately after the intervention to assess its effects. All participants engaged in daily rehabilitation training at the institution, utilizing methods such as Applied Behavior Analysis (ABA), Floor Time, RDI, and TEACCH. Specifically, participants in the cTBS group received cTBS stimulation in addition to their routine rehabilitation training. Similarly, those in the BCTP group participated in BCTP interventions alongside their regular sessions. The CG group, however, only participated in the routine rehabilitation training without additional interventions. The CIG group underwent a combination of both cTBS and BCTP interventions during their regular training schedule, receiving cTBS stimulation in the morning and BCTP intervention in the afternoon. This structured approach ensured that all interventions were consistent and comparable across different groups.

Control variable

In the preliminary assessment, demographic data (age, gender) of participants were collected, as detailed in Table 3. The Childhood Autism Rating Scale (CARS) (Schopler et al., 1980) and the Wechsler Intelligence Scale for Children, Fourth Edition (WISC-IV), were used to determine the presence and severity of autism as well as the IQ level. Previous studies have shown links between executive function[9], sleep[10], repetitive stereotyped behaviors[11], social interactions[12], and the diets of children with ASD. To control for these confounding variables, we used The Child Executive Functioning Inventory, The Children's Sleep Habits Questionnaire (CSHQ), the Repetitive Behavior Score (RBS-R), and the Social Responsiveness Scale, Second Edition (SRS-2) for assessments. The CARS assessment was conducted by a hospital-based medical professional, while the WISC-IV assessment was conducted by a qualified experimenter. The SRS-2, RBS-R, CSHQ, and CHEXI assessments were completed by the participants' parents.

Primary Outcomes

The results of this study are reported using the Children's Eating Behavior Questionnaire (CEBQ), Chinese version, which was culturally adapted from the original English version and has been widely validated in China with good reliability and validity (Cronbach's $\alpha > 0.7$). The CEBQ consists of 35 items across eight dimensions. These dimensions assess various aspects of children's eating behaviors through eight subscales: Food Responsiveness (FR), Enjoyment of Food (EF), Emotional Over-Eating (EOE), Desire to Drink (DD), Satiety Responsiveness (SR), Slowness in Eating (SE), Emotional Under-Eating (EUE), and Food Fussiness (FF). Each dimension includes 3-6 items, with responses ranging from "Never" to "Always," scored from 1 to 5. Items Q3, Q4, Q10, Q16, and Q32 are scored inversely. The score for each dimension is the average of its items, with higher scores indicating more frequent eating behaviors in that dimension.

References

1. Baraskewich J, von Ranson KM, McCrimmon A, McMorris CA. Feeding and eating problems in children and adolescents with autism: A scoping review. Autism. 2021 Aug;25(6):1505–19.
2. Ahearn WH, Castine T, Nault K, Green G. An assessment of food acceptance in children with autism or pervasive developmental disorder-not otherwise specified. J Autism Dev Disord. 2001 Oct;31(5):505–11.
3. Harris HA, Bowling A, Santos S, Greaves-Lord K, Jansen PW. Child ADHD and autistic traits, eating behaviours and weight: A population-based study. Pediatr Obes. 2022 Nov;17(11):e12951.
4. Nabors L, Overstreet A, Carnahan C, Ayers K. Evaluation of a pilot healthy eating and exercise program for young adults with autism spectrum disorder and intellectual disabilities. Adv Neurodev Disord. 2021 Dec 1;5(4):413–30.
5. Cai KL, Wang JG, Liu ZM, Zhu LN, Xiong X, Klich S, et al. Mini-Basketball Training Program Improves Physical Fitness and Social Communication in Preschool Children with Autism Spectrum Disorders. J Hum Kinet. 2020 Jul;73:267–78.
6. Wang S, Chen D, Yang Y, Zhu L, Xiong X, Chen A. Effectiveness of physical activity interventions for core symptoms of autism spectrum disorder: A systematic review and meta-analysis. Autism Res. 2023 Sep;16(9):1811–24.
7. Annesi JJ. Supported exercise improves controlled eating and weight through its effects on psychosocial factors: Extending a systematic research program toward treatment development. The Permanente Journal. 2012 Mar;16(1):7–18.
8. Lowe C. The effects of continuous theta burst stimulation (cTBS) to the left dorsolateral prefrontal cortex on executive control resources, subjective food cravings, and the consumption of appetitive snack foods. 2014 Jan 24 [cited 2025 Feb 7]; Available from: http://hdl.handle.net/10012/8202
9. Cepni AB, Power TG, Ledoux TA, Vollrath K, Hughes SO. The longitudinal relationship between diet quality and executive functioning development of hispanic preschoolers in houston, texas. J Acad Nutr Diet. 2025 Mar;125(3):386-395.e1.
10. Ogilvie RP, Lutsey PL, Widome R, Laska MN, Larson N, Neumark-Sztainer D. Sleep indices and eating behaviours in young adults: Findings from project EAT. Public Health Nutrition. 2018 Mar;21(4):689–701.
11. Sharp WG, Berry RC, McCracken C, Nuhu NN, Marvel E, Saulnier CA, et al. Feeding problems and nutrient intake in children with autism spectrum disorders: A meta-analysis and comprehensive review of the literature. J Autism Dev Disord. 2013 Sep 1;43(9):2159–73.

12. Kerr-Gaffney J, Harrison A, Tchanturia K. The social responsiveness scale is an efficient screening tool for autism spectrum disorder traits in adults with anorexia nervosa. European Eating Disorders Review. 2020;28(4):433–44.
